# Autistic and non-autistic adults use discourse context to determine a speaker’s intention to request

**DOI:** 10.1007/s10339-024-01229-6

**Published:** 2024-09-24

**Authors:** Faith Frost, Marisa Nagano, Emily Zane

**Affiliations:** 1 Communication Sciences and Disorders at James Madison University, Health and Behavioral Studies Building, 235 Martin Luther King Jr. Way, Rm. 1068, Harrisonburg, VA 22801 USA; 2 Independent Researcher, New York, NY USA

**Keywords:** Autism, Indirect requests, Mentalizing, Pragmatics

## Abstract

The current study focuses on how autistic adults utilize context to determine whether ambiguous utterances (e.g., “I’m thirsty”) are intended as indirect requests or as literal comment/questions. Two questions are addressed: (1) How do autistic adults compare to neurotypical adults in using context to interpret an utterance’s intention as either literal or a request? (2) What cognitive mechanisms correlate with indirect request interpretation, and are these different for participants in each group? Twenty-six autistic and 26 neurotypical college students participated, engaging in an online experiment where they read narratives that ended with utterances open to literal or request interpretations, based on context. After each narrative, participants selected the best paraphrase of the utterance from two options, literal versus request. Following this task, participants completed two mentalizing measures (a false belief and emotion-identification task) and several executive functioning tests. The best model for predicting paraphrase choice included scores on the emotion-identification task and context as main effects, along with the interaction between both. Participants with higher emotion-identification test scores were more likely to provide correct paraphrases. Models including group as a main effect and/or interaction were not better at fitting the data, nor were any models that included executive functioning measures as main effects or interactions. Emotion-identification test scores, but not autism diagnosis, predict how adults infer whether an utterance is a request. Findings suggest that autistic adults use context similarly to neurotypical adults when interpreting requests, and that similar processes underlie performance for each group.

## Introduction

English speakers can choose from several utterance types when they want to perform a directive which is a speech act wherein the speaker’s intention is to command or request (Searle 1975). These include imperatives (e.g., “Turn on the heater”.), interrogatives (e.g., “Isn’t it cold in here?”) and declaratives (e.g., “It’s cold in here”.). The latter two forms, interrogatives and declaratives, are referred to as “indirect requests”, since, unlike imperatives, the form of the locutionary act (i.e., the linguistic structure) belies its illocutionary force (i.e., the intended meaning). Although imperatives more straightforwardly convey a speaker’s intention to command or request, English speakers commonly opt for indirect requests, instead, as they are perceived as being more polite (Brown and Levinson 1987; Lakoff 1973). However, being polite through indirectness presents a potential processing challenge, as the listener must recognize that the utterance’s form belies its intention to direct. If a listener does not recognize this, they may respond in an undesirable way. For example, if a speaker asks, “Isn’t it cold in here?”, a listener may answer the question (e.g., “Yes, it is”.) rather than perform the intended action (i.e., turn on the heater). Thus, a listener’s ability to recognize that non-imperative forms (i.e., interrogatives and declaratives) can be used to convey an imperative intention is important for successful communication.

Some types of indirect requests are easier to interpret than others. For example, certain indirect request forms introduced by a modal (e.g., “Can you turn on the heater?”)—labeled embedded^1^ imperatives in an influential framework by Ervin-Tripp (1976) since they contain the structural imperative (i.e., “turn on the heater”)—have traditionally been referred to as “conventionalized” indirect requests (Ackerman 1978; Gibbs 1986). This label reflects the fact that listeners seem to automatically interpret embedded imperatives beginning with “can” or “could” as requests rather than yes/no questions when they are presented in a context that allows for a directive interpretation (Gibbs 1986). In support of the notion that embedded imperatives are easily recognized as requests, research finds that typically developing children successfully understand embedded imperatives as early as age two (Babelot and Marcos 1999; Bucciarelli et al. 2003; Shatz 1978a; b). There are other types of indirect requests that may pose a greater challenge for successful comprehension. For example, speakers sometimes produce utterances with embedded imperatives but no initial modal (e.g., “I wish someone would turn on the heater”.), other times speakers use declaratives or interrogatives that do not include the structural imperative in the surface form at all (e.g., “I wish the heater was on”.), and sometimes they even fully exclude all parts of the underlying imperative (e.g., “It’s cold in here”.). These forms have been conceptualized as “non-conventionalized” requests given that they employ utterances that are not routinely directive (Ackerman 1978). The latter two types—in which the structural imperative does not appear in the surface form at all—arguably presents the most challenge to listeners. Ervin-Tripp’s framework referred to such forms as “hints” or “question directives”, depending on whether the surface form is declarative or interrogative, respectively (Ervin-Tripp 1976; 1977). For the sake of simplicity, we will refer to any type of indirect request that does not involve most or all the structural imperative as “hints”, regardless of whether their surface form is a question or statement.

### Indirect requests and theory of mind

Given that the default interpretation of hints is not necessarily imperative, listeners must rely on context and inferencing skills to determine the speaker’s intended meaning. For example: A driver and a passenger are in a car that has a broken heating system, and the passenger says, “It’s cold in here”. The interpretation of this utterance depends on the background knowledge each interlocuter possesses about the other, including their understanding of the other person’s mental state (Trott and Bergen 2018; 2020). If the passenger knows the heater is broken (and the driver knows the passenger knows this), “it’s cold in here” is likely intended as a comment, and the driver will interpret it as such. However, if the passenger is unaware of the heater’s status (and the driver knows they are unaware), the same utterance could be intended and interpreted as a request. Thus, a listener’s successful interpretation of whether a speaker means to hint or to comment is wholly dependent on their understanding of the speaker’s mental representation. Accordingly, research has shown that successful interpretation of hints develops alongside theory of mind (ToM) skills. ToM describes the ability to infer the mental states of others. In laboratory settings, ToM ability is often assessed through inferencing tasks like comprehending false beliefs. Research finds that ToM skills are developed gradually in typically developing children, with certain ToM subskills, like understanding false beliefs, not emerging until children are between the ages of four and six years (Wimmer and Perner 1983). Similarly, children start understanding hints around this age, although not perfectly (Ackerman 1978; Bernicot et al. 2007; Bernicot and Legros 1987; Bucciarelli et al. 2003; De Mulder 2015; Elrod 1987; Ledbetter and Dent 1988; Leonard et al. 1978; Liebling 1988; Spekman and Roth 1985).

Research on neurotypical (NT) adults has also provided evidence that ToM skills are related to hint comprehension (Trott and Bergen 2018; 2020; van Ackeren et al. 2012). Trott and Bergen (2018) tested NT adults on their ability to determine whether an utterance was a comment/question or a hint, depending on the speaker’s state of mind in a given context. In their experiment, participants read short narratives that ended with an utterance that could be interpreted as a comment/question or a request. Narratives were manipulated so that speakers were either aware or unaware of circumstances making a request in/felicitous, similar to the car heater example in the previous paragraph. After reading each narrative, participants selected the best paraphrase of the utterance from two options: literal (i.e., comment/question) versus request. It was found that participants who performed less accurately on the indirect request task had lower mentalizing scores, leading authors to conclude that ToM skills underlie the successful interpretation of hints.

The link between ToM and hint comprehension could suggest that autistic^2^ listeners would show differences compared to non-autistic peers in their interpretation of hints since differences in ToM have long been touted as a feature of autism (Baron-Cohen 2000; Baron-Cohen et al. 1985; cf. Milton 2012). Previous work does indeed provide evidence for a relationship between autism, ToM, and non-literal language interpretation. The “Strange Stories” test, for example, uses vignettes to assess individuals’ comprehension of several types of non-literal language, including irony, lying, metaphor, and persuasion (Happé 1994). In numerous studies, research has found that autistic groups perform less well on this measure compared to non-autistic groups, and that group differences in performance correlate with other ToM measures, like false belief tasks (e.g., Happé 1994; Jolliffe and Baron-Cohen 1999; White et al. 2009). In fact, the “Strange Stories” task has *itself* been touted as a ToM task (e.g., Happé, 1994). They make this argument, not only because scores correlate with performance on other ToM measures, but also because successful non-literal language interpretation, and therefore task performance, is arguably dependent on participants’ understanding of a speaker’s mental state in a given discourse context.

Just like the “Strange Stories” vignettes, Trott and Bergen (2018)’s narratives specifically manipulate discourse context so that a speaker’s mental state in a given context can be used to guide interpretation of the speaker’s intention to literally comment/question or non-literally hint. As such, there are reasons to suspect differences in autistic versus non-autistic performance on such a task. However, it is difficult to confirm this hypothesis based on existing research, as most previous work has focused on how autistic people interpret conventionalized, embedded requests rather than hints.

### Indirect request comprehension in autism

While there is limited research on interpretation of hints, research exploring how autistic individuals interpret conventionalized, embedded requests has yielded fairly consistent results, with both autistic children and adults successfully interpreting these forms (Deliens et al. 2018; Kissine et al. 2012; Ozonoff and Miller 1996; Paul and Cohen 1985). The earliest of these studies, conducted by Paul and Cohen (1985), found that autistic adults with a mental age of 4–7 years were successful at recognizing that interrogatives including an embedded imperative (e.g., “Can you color the circle blue?”) can function as requests. Since then, these results have been replicated with both autistic children (Kissine et al. 2012) and autistic adults with average IQ scores (Deliens et al. 2018; Ozonoff and Miller 1996).

While this literature does establish that autistic people can recognize embedded requests as requests, there is also some evidence that autistic people might struggle to utilize context to determine that such forms are *not* meant to request. In Ozonoff and Miller (1996), autistic participants were more likely to interpret utterances beginning with, “Can you…?” as requests, no matter the context, meaning that they interpreted these forms as requests even when the context biased a literal interpretation. These authors interpreted their findings as indicating the autistic participants have difficulty utilizing context to determine speaker meaning. Deliens and colleagues (2018) found that autistic participants interpreted embedded request questions beginning with “Can you…?” as requests similarly to non-autistic counterparts. However, autistic participants also interpreted “non-conventionalized requests” (questions beginning with, “Is it possible…?”) as requests, and they did this significantly more often than NT participants. Together, the Ozonoff and Miller (1996) and Deliens and colleagues (2018) findings may suggest that autistic individuals base their interpretation of requests on very specific features of the surface form (i.e., the inclusion of an embedded imperative in an interrogative), while ignoring factors external to that embedded request (i.e., whether it is preceded by a modal or “is it possible”) and external to the utterance itself (i.e., discourse context). Such an interpretation corresponds with the Weak Central Coherence (WCC) theory, which hypothesizes that autistic individuals tend to focus more on the specific and local (in this case a specific utterance’s form), rather than integrating information from the surrounding, global context to interpret meaning (Happé and Frith 2006). Studying how autistic people interpret hints, which do not include an embedded imperative and which do not support a default request interpretation, could shed light on how autistic individuals utilize context to interpret speaker meaning, since the successful interpretation of a hint can *only* depend on global context.

As far as we are aware, research on autistic people’s understanding of hints has only included children, not adults. MacKay and Shaw (2004) compared how autistic and NT children (ages 8–11) interpreted hints by having participants listen to short stories that ended with an utterance with an ambiguous intention, such as, “That cake looks delicious”. Participants had to then explain what the speaker meant. In all trials, these utterances were intended to be hints; that is, the context made it clear that the speaker’s intention was to request, not to comment. Results indicated that autistic and NT children were similarly able to explain speaker intention, suggesting they all recognized that the function of the utterances was to request. However, because all target utterances were intended as requests, results do not shed light on how autistic children use context to interpret the same utterance in different ways. For example, in certain contexts (e.g., at a party) the utterance, “That cake looks delicious”, can be interpreted as a request, while in other contexts (e.g., watching a baking show) it can only be interpreted literally, as a comment. Thus, it is possible that MacKay and Shaw (2004)’s findings mirror those from Ozonoff and Miller (1996), discussed earlier, where autistic individuals interpreted ambiguous utterances as requests, regardless of context. If so, we predict that group differences would be discovered if participants had to incorporate discourse context—particularly context manipulating mental states of the speaker and listener—in order to determine whether the *same* utterance is intended as a request or not in a given situation.

Kissine and colleagues (2015) incorporated just this manipulation by recording children’s responses to utterances that were presented in both directive (i.e., hint) and literal (i.e., comment) contexts. For this study, participants engaged with a Mr. Potato Head toy, with two experimenters present—one interacting directly with the child and the other nearby. In the directive context, the interacting experimenter said to the child, “Oh, he has no hat!” as a request to put a hat on the toy. In the literal context, the same utterance (“Oh, he has no hat!”) was again produced by the experimenter while the child engaged with Mr. Potato Head, but the discourse context biased a non-request interpretation, by manipulating both the utterance’s addressee (the comment was directed towards another adult in the room) and the utterance’s referent (the comment was made about a character in picture). Results indicated that autistic children were successful at responding to the intention of these utterances, complying with the hint in the directive context and refraining from interpreting it as a request in the literal context. The authors concluded that their findings not only confirmed those of MacKay and Shaw (2004), showing that autistic children can interpret hints, but also built on those findings by revealing that autistic children can use discourse context to distinguish a speaker’s intention. However, there were some aspects of Kissine and colleagues (2015) that make the interpretation of findings less straightforward. The literal context’s adjustments to addressee and referent provided multiple cues to participants that the statement was not intended as a request, making it unclear which cue children relied on for interpretation. The fact that children got credit for correctly “complying” with a literal intention by *not* doing something (i.e., not putting a hat on Mr. Potato Head) also complicates interpretation of findings. It is possible that children who said or did nothing in response to a “comment” may have simply been ignoring the utterance altogether, a behavior that seems perfectly legitimate when the speaker is talking to someone else. Therefore, a child might not have attempted to interpret the utterance one way or another and could still get credit for interpreting it successfully. To obtain conclusive evidence that autistic children can effectively utilize context to determine speaker intention, it is necessary that both the non-literal (request) and literal (comment) forms are directed towards the same person, and that a participant’s interpretation can be unambiguously reflected by their response, rather than a lack of response.

A more recent study by Marocchini and colleagues (2021) aimed to investigate the relationship between ToM and indirect request interpretation in both NT and autistic children (ages 9–12). To assess this, participants completed first- and second-order ToM tests, in addition to an indirect request task. For their indirect request task, children were presented with a drawing and asked to help the experimenter (who had seen the picture before, but could not see it during the task) to recreate it. Experimenters used both imperatives (e.g., “Tell me what color the grass is”.) and hints (e.g., “I don’t remember the color of the grass”.) to elicit information from participants. Overall results indicated no main effect of group, suggesting that autistic children were as successful as their NT peers at interpreting indirect requests. This is noteworthy because the NT group had significantly higher scores on the ToM tests. This contradicted the authors’ initial hypothesis that ToM scores would correspond with indirect request compliance, leading them to conclude that while NT children may rely on ToM to interpret hints, autistic children use alternative interpretative strategies. The authors suggest that autistic children may use certain linguistic cues, such as the phrase *color of the grass,* to guide them into producing a correct response, even if they do not truly recognize the speaker’s intention.

While Marocchini and colleagues (2021) offers some insights into the relationship between ToM and hint interpretation in autistic children, it also has some limitations. First, similar to MacKay and Shaw (2004), Marocchini et al. did not vary the context nor the intent of the target utterances, so that all items were only presented once, and always with a directive intention. Thus, we cannot determine how groups would use context to determine whether a given utterance was meant as a request or a literal comment/question. Second, as in Kissine and colleagues (2015), Marocchini et al. considered participants’ non-responses as meaningfully signaling the child’s understating. In the Marocchini et al. study, a child’s lack of response was interpreted as children *misunderstanding* the speaker’s intention to request, whereas in Kissine et al. (2015), non-responses to comments were interpreted as children *understanding* them. As we outlined above, there are reasons children may remain silent that have little to no bearing on whether they recognize a speaker’s intent. Finally, since all the research on hint interpretation in autism has included children (and not adults), it is difficult to tease apart development from diagnostic group. That is, group differences may merely reflect developmental trends that disappear when participants reach adulthood.

### The current study

The current study addresses gaps identified in previous research by examining how autistic adults use discourse context to determine whether an utterance is intended to request. Specifically, we focus on the interpretation of hints, given that these forms do not encourage a default request interpretation the way that conventionalized embedded requests do. While there is a substantial amount of evidence indicating that autistic individuals are successful at interpreting embedded requests (Deliens et al. 2018; Kissine et al. 2012; Ozonoff and Miller 1996), there is no consensus on how autistic individuals (particularly adults) interpret requests that do not include the form and/or semantic components of the underlying imperative.

As such, the current design replicates Trott and Bergen (2018; 2020)’s studies on NT adults, where participants read short narratives that end with a speaker producing an ambiguous utterance, and then participants select an appropriate paraphrase for the utterance. Because participants must explicitly provide an interpretation for each utterance, we avoid the pitfalls of using participants’ response behavior (or lack thereof) as a window into their understanding of speaker intention.

Two questions will be examined: (1) How do autistic adults compare to NT adults in using discourse context to interpret an utterance’s intention as either a request or a comment/question? (2) What cognitive mechanisms correlate with performance on indirect request interpretation, and are these different for participants in each group? Regarding the first question, it is difficult to form a hypothesis based on the existing literature. While results from Ozonoff and Miller (1996) suggest that autistic adults may show differences in using context to guide the interpretation of conventionalized requests versus literal questions, Kissine and colleagues (2015) found that autistic children adaptably used context to distinguish hints from comments. Because we suspect that findings in the latter study were influenced by the fact that children had multiple cues to drive interpretation (including changes in the addressee), we tentatively predict that autistic adults will show less sensitivity to discourse context, specifically related to whether a speaker is aware of information making a request infelicitous, and will therefore have difficulty determining whether a speaker intends to request or not in a given scenario. This hypothesis is also motivated by two other factors. First, Trott and Bergen (2018; 2020) found that ToM scores correlated with performance on their indirect request measure, and we predict that autistic participants will score lower on ToM measures in the current study. Second, the WCC theory suggests that autistic participants may show differences in how they utilize the global discourse context to determine the meaning of a given sentence (Happé and Frith 2006).

The second research question explores the cognitive constructs that underlie indirect request interpretation in autistic and NT adults, and whether different mechanisms may be involved for each group. One of the cognitive constructs we measure is ToM, since previous research has suggested a link between ToM and indirect request comprehension for NT adults and some NT children (Marocchini et al. 2021; Trott and Bergen 2018; 2020; van Ackeren et al. 2012). Based on their findings, we hypothesize that ToM scores will predict indirect request interpretation performance for NT adults who participate in the current study. Regarding autistic adults, it is again difficult to make a prediction. Marocchini and colleagues (2021) found that ToM scores did not predict performance on an indirect request task for autistic children, suggesting that autistic individuals may use strategies aside ToM to interpret indirect requests correctly. However, this finding may only reflect how autistic *children*–not autistic adults–perform such tasks. Although autistic children tend to score lower than NT children on ToM tests, these differences decrease with development (Happé, 1995; Scheeren et al. 2013; Steele et al. 2003). In fact, some autistic adults score similarly to NT adults on certain ToM measures (see Gernsbacher and Yergeau 2019 for a review). Therefore, while autistic children may rely less on ToM to interpret requests, autistic adults may use similar strategies to NTs.

In addition to ToM, we also explore how executive functioning (EF) relates to indirect request interpretation for both groups. EF refers to a set of cognitive processes that are necessary for managing thought and behavior. Core subcomponents include inhibition, attention shifting, and working memory (Miyake et al. 2000). Because some previous research has suggested that indirect request interpretation may function independently of ToM for autistic children (Kissine et al. 2015; Marocchini et al. 2021), it is possible that other cognitive mechanisms may underlie their interpretation. We offer EF as one possibility, since the ability to incorporate the surrounding/preceding discourse into the interpretation of a speaker intention arguably relies on attentional allocation to relevant aspects of the discourse, memory of those relevant aspects, and inhibition of irrelevant information. Inhibition is also likely involved in determining that a sentence is meant as a request rather than a literal comment/request (and vice versa), since one must inhibit the competing interpretation. In support of the fact that indirect request can involve EF, at least for certain populations, Champagne-Lavau and Joanette (2009) conducted a study examining the roles of ToM and EF in interpreting indirect requests in adults with right-hemisphere brain damage. While EF scores alone did not predict performance on indirect request measures, the authors found that a combination of ToM and EF best accounted for participant performance. We therefore hypothesize that individuals who score lower on EF tasks assessing inhibition, attention shifting, and working memory will have less accurate interpretations.

## Methods

### Participants

English-speaking college students were recruited via university bulk email. This study was approved by the Institutional Review Board at James Madison University; all participants provided email consent to participate in research.

The autistic (AUT) group consisted of 26 young adults (age range = 18–25 years, mean age = 19.9). In the AUT group, 14 adults reported being assigned the female sex at birth, and 12 adults reported being assigned the male sex at birth. Current gender identities included: transgender woman (3.8%), transgender man (3.8%), agender (3.8%), nonbinary (23.1%), cisgender woman (23.1%), cisgender man (38.5%), and 3.8% of autistic participants identified as other (not listed). The racial and ethnic backgrounds of the AUT group included: Native American (3.8%), Black or African American (7.7%), Latinx (15.4%), White (61.5%), and 11.5% identified as being two or more races.

The AUT group included both individuals who had been officially diagnosed as autistic/having autism (*n* = 12; 6 males), as well as individuals who were self-diagnosed (*n* = 14; 6 males). We chose to include individuals who diagnosed themselves as autistic due to the protracted diagnosis of autism in adulthood (Lewis 2017). Given research demonstrating that some autistic individuals may show fewer characteristics of autism as they grow older (Howlin and Magiati 2017; Riglin et al. 2021), the Autism Quotient (AQ; Baron-Cohen et al. 2001a, b) was administered to determine each participants’ current level of autistic features. Overall, autistic participants had an average AQ score of 31.4 (see Table 1). Individuals with official diagnoses had an average AQ score of 29.0 (ranging from 10 to 37), while individuals who self-diagnosed had an average AQ score of 33.4 (ranging from 21 to 44). Additionally, 10 autistic participants (1 male; 9 females) reported co-occurring diagnoses of ADHD. These individuals had an average AQ score of 32.5 (ranging from 27 to 38).

**Table 1:** Participant characteristics

	AUT (*n* = 26)	NT (*n* = 26)	*t*	*p*
Age	19.9 (1.9)	19.7 (1.7)	− 1.07	0.28
Autism Quotient	31.4 (6.5)	17.8 (5.4)	− 23.20	< 0.001**
SAAB	14f:12m	15f:11 m	− 0.79	0.43

*SAAB* Sex assigned at birth. *p < .01, ** *p* < .001

The neurotypical (NT) group consisted of 26 young adults (age range = 18–24 years, mean age = 19.7). Exclusion criteria for the neurotypical group included having received a diagnosis of any of the following: ADHD/ADD, autism/ASD, deafness or hearing loss, dyslexia or reading disorder, intellectual impairment, language disorder, learning disorder, schizophrenia, seizure disorder, or traumatic brain injury. In the NT group, 15 reported being assigned the female sex at birth, and 11 reported being assigned the male sex at birth. Current gender identities included: transgender woman (3.8%), nonbinary (7.7%), cisgender man (38.5%), and cisgender woman (50.0%). The racial and ethnic backgrounds of the NT participants included: Asian (7.7%), Black or African American (7.7%), Latinx (7.7%), White (65.4%), and 11.5% identified as being two or more races. The average AQ score for the NT participants was 17.8 (ranging from 10 to 29).

Because all participants were currently pursuing either bachelor’s or master’s degrees at an institution of higher education, we assumed them to have functional language skills in at least typical ranges. However, as the indirect request task specifically required participants to read and understand short passages, we included passing scores on a 5-question reading comprehension screener adapted from the Test of Reading Comprehension-Fourth Edition (Brown et al. 2009) as an inclusion criterion.

Initially, 55 participants were recruited for the experiment, but only 52 were included in the data analysis. Two participants (1 autistic and 1 NT) were excluded due to incomplete experiments, and one NT participant was excluded for failing the reading comprehension screener.

## Materials

### Indirect request task

The stimuli for the indirect request task consisted of eight pairs of short narratives, originally created by Trott and Bergen (2018), and minorly adapted by the current authors after feedback from pilot participants. Each narrative pair described the same scenario, in which the participant (addressed as “you”) interacts with a character in the story who ultimately produces an utterance that could be interpreted as either a comment/question or a request, depending on discourse context (Appendix A). As in Trott and Bergen (2018), the manipulation within each narrative pair involved whether speaker was aware of circumstances making a request in/felicitous. For example: You and your friend are riding in a car, and your friend says, “I’m cold”. Depending on whether your friend knows the car’s heater is broken—information that is explicitly supplied in the narrative—the final utterance could be interpreted as a comment or a request. Thus, just as in Trott and Bergen (2018), there were two versions of each narrative: (1) A *Speaker Aware* version, in which the speaker knew information making a request infelicitous (e.g., the car’s heater is broken); (2) A *Speaker Unaware* version, when it was clear that the speaker was not privy to that information.

Participants were randomly assigned to one of two lists, counterbalanced for which version of each narrative they read. Each participant read eight passages, presented in a random order, with an equal number of narratives in the Speaker Aware and Speaker Unaware conditions (four in each). After reading each narrative, participants were required to choose the best paraphrase of the speaker’s utterance from two options. For convenience, we refer to the paraphrase choices as “correct” or “incorrect” in the Results section, although neither paraphrase is inherently right or wrong. One paraphrase option represents a literal interpretation of the utterance (e.g., *I’m really cold; it’s too bad the heater is broken*.), while the other is a request, formulated as a modal-question indirect request (e.g., *Could you turn on the heater?*). The paraphrase judgement options were presented in random order. The experiment software automatically recorded the amount of time participants spent on each task.

### Theory of mind measures

Two measures were administered to assess participants’ ToM abilities: a false belief task and the Reading the Mind in the Eyes test (RME; Baron-Cohen et al. 2001a, 2006b). The false belief task was administered prior to the indirect request task, while the RME was administered following the indirect request task.

It is worth noting that these measures differed from the ToM measure used in Trott and Bergen (2018; 2020). The measure utilized in their experiment, called the Short Story Task (SST; Dodell-Feder et al. 2013), required participants to answer mentalizing and comprehension questions based on a short story by Ernest Hemingway. Mentalizing was measured by observing participants’ spontaneous and prompted use of mental state terms. The authors labeled spontaneous use as “mental state inferencing” and prompted use as “mental state reasoning”. It was found that only mental state reasoning was predictive for the indirect request task. However, they found that mental state reasoning scores were highly correlated with reading (narrative) comprehension scores, making it difficult to know which construct was underlying performance on the request task.

To help tease apart the contribution of narrative comprehension and ToM on participants’ performances on the indirect request task, we decided to include the RME. The RME assesses mental state recognition by requiring participants to identify mental states based on visual information from the eyes, and thereby not involving narrative comprehension. We also elected to replace the SST with a false belief task that may also rely less on complex narrative comprehension than participants’ interpretation of a passage written by Ernest Hemingway. For the false belief task, participants were presented with three short stories and asked 1–2 false belief questions after each (Appendix B). The false belief questions tested both first- and second-order ToM, assessing how participants attribute mental states to characters and use that information to predict other mental states and actions. The decision to use the false belief task, rather than the SST, was also motivated by the fact that participants in the current study were administered the AQ and several EF tasks, along with the indirect request task. Given that the SST is a lengthy measure, we believed that replacing it with a shorter measure would better ensure task engagement and completion.

### Executive functioning measures

Three EF tasks were administered to evaluate inhibition, attention shifting, and working memory. Inhibition was assessed using a flanker task (Eriksen and Eriksen 1974), attention shifting was measured through the Wisconsin Card Sorting Task (WCST; Berg 1948), and working memory was evaluated using an n-back task.

### Procedure

The experiment was conducted online, implement using jsPsych (de Leeuw 2015). Upon completing the demographic survey and reading comprehension screener, participants engaged in the false belief task. Next, participants completed the indirect request task. Then, the RME was administered as an additional ToM measure. Following the RME, participants responded to the AQ. Finally, the participants were administered the three EF tasks. The experiment took an average of 40 min to complete, and participants received compensation ranging from $15 to $20 for their participation.

### Statistical analysis

All analyses were performed in R (R Core Team 2023). Generalized linear mixed-effects models were run using the *lme4* package (Bates et al. 2015).

## Results

### Interpretation data

First, we tested whether the narrative context (speaker aware vs. speaker unaware) predicted participants’ interpretations. To do this, we built a simple model with Interpretation (literal vs. request) as the dependent variable, Condition (speaker aware vs. speaker unaware) as a fixed effect, and a random intercept for Item. Results from the simple model revealed that Condition significantly predicts whether participants choose a literal or request interpretation [$\upbeta$ = 3.77, *SE* = 0.31, *p* < .001]. All models described in the following contain these same effects—a fixed effect for Condition and a random intercept for Item—with additional fixed effects, and then we compare the fit of those models to the original, reduced model with Condition as the only fixed effect.

Our first research question focused on comparing groups’ ability to utilize speaker awareness to interpret an utterance as either a request or a comment/question. To do this, we added Group as a fixed effect to the null model, along with the interaction between Group and Condition. We then compared this model to the reduced model. Results revealed that adding Group and the interaction between Group and Condition did not improve model fit compared to the original, reduced model [χ^2^(2) = 1.32, *p* = 0.52]. Table 2 presents the proportion of literal and request responses by the two groups in each condition.

**Table 2:** Proportion of responses by group per condition

Condition	AUT		NT	
	Literal (%)	Request (%)	Literal (%)	Request (%)
Speaker aware	85.6	14.4	89.4	10.6
Speaker unaware	13.5	86.5	17.3	82.7

In both conditions, AUT adults and NT adults exhibited similar response patterns. Specifically, in the Speaker Aware condition, both groups more often selected a literal (comment/question) interpretation, while in the Speaker Unaware condition, both groups more often selected a request interpretation [AUT: χ^2^(1) = 108.18, *p* < 0.001; NT: χ^2^(1) = 108.67, *p* < 0.001].

Given that groups were defined on participants’ disclosure that they were autistic (and included participants who were self-diagnosed) we conducted analysis examining how one’s level of autistic features, as measured by the AQ, correlate with one’s performance on the indirect request task. To do this, we added AQ as a fixed effect to the null model, along with the interaction between AQ and Condition. We then compared this model to the reduced model. Results revealed that adding AQ and the interaction between AQ and Condition did not improve model fit compared to the original, reduced model [χ^2^(2) = 2.70, *p* = 0.26].

Because analyses involving both Group and AQ resulted in null findings, we ran post-hoc Bayesian analyses to provide evidence in favor of the null model. Bayesian generalized linear mixed effect models predicting response type (Request = 1, Literal = 0) were conducted in R using the brms package (Bürkner 2017). As in the initial frequentist analyses, random factors included Item and Participant, while fixed factors of Condition and Group/AQ were added and compared to smaller models using the bayes_factor function in brms. Models used the brms default for priors (improper flat priors), chains (4), and iterations (2000). Family was set to Bernoulli with a logit link.

Bayes factor analysis provided extreme evidence for the model with the fixed effect of Condition (M_C_) over the null model with random effects alone (M_0_) (BF_C0_ > 100). Bayes factor analyses provided anecdotal evidence for the null model with random effects alone (M_0_) over a model that added a fixed effect of a binary Group predictor (M_G_) (BF_GO_ = 0.626), and very strong evidence for the null model (M_0_) over a model that added a fixed effect of AQ (M_AQ_) (BF_0AQ_ = 0.027) (using the cutoffs from Jeffreys 1939). As such, the Bayesian analysis provides strong evidence for the effect of Condition over a null model, strong evidence for the null model over AQ, and some evidence for the null model over a binary Group predictor.

### Cognitive measures

Our second research question focused on assessing what cognitive mechanisms predict performance on indirect request interpretation. Table 3 reports the groups’ mean scores on both ToM tasks and the three EF tasks.

**Table 3:** Mean score (SD)/highest possible score for each group in the tasks assessing ToM and EF

	AUT	NT	*t*	*p*
False belief	3.81 (.40)/4	3.96 (.19)/4	5.05	< 0.001**
RME	26.46 (3.51)/36	26.85 (3.65)/36	1.10	0.27
Flanker	29.62 (3.23)/30	29.50 (4.64)/30	− 0.29	0.77
WCST	45.23 (6.22)/60	46.77 (5.78)/60	2.61	0.009*
N-Back	25.23 (3.45)/30	22.73 (4.03)/30	− 6.79	< 0.001**

*p < .01, **p < .001

Significant group differences were found for the false belief task, the WCST (i.e., attention shifting), and the n-back task (i.e., working memory). No significant differences were found between the groups on the RME or the flanker task (i.e., inhibition). While participants exhibited some differences in their performances on these measures, the fundamental aim of our second research question was to determine whether any of these measures have predictive value for individuals’ performance.

We began with the first ToM test scores, the false belief task, by adding in scores from this task as fixed effects, along with the interaction between Condition and False Belief scores. We then compared this model to the reduced model, which was the as-yet best-fitting model. Results revealed that adding scores from the false belief task, along with the interaction, did not improve model fit compared to the reduced model [χ^2^(2) = 0.46, *p* = 0.79].

We then moved on to our second ToM measure, the RME. We added RME scores as fixed effect to the as-yet best-fitting model, along with the interaction between Condition and RME scores. We then compared this model to the reduced model. Results revealed that adding RME scores, along with the interaction between RME and Condition, significantly improved model fit, as compared to the model that included Condition as the only fixed effect [χ^2^(2) = 12.16, *p* < 0.01] (Table 4).

**Table 4:** Model of best fit

Predictors (fixed effects)	Parameter estimates		Wald’s test		Odds ratio
	$\upbeta$	*SE*	*z*	*p* ($\upbeta$ = 0)	
Condition	− 3.38	2.12	− 1.59	0.11	0.03
RME	− 0.11	0.06	− 1.90	0.06	0.89
Condition: RME	0.28	0.08	3.33	< 0.001**	1.32

*p < .01, **p < .001

**Fig. 1:**
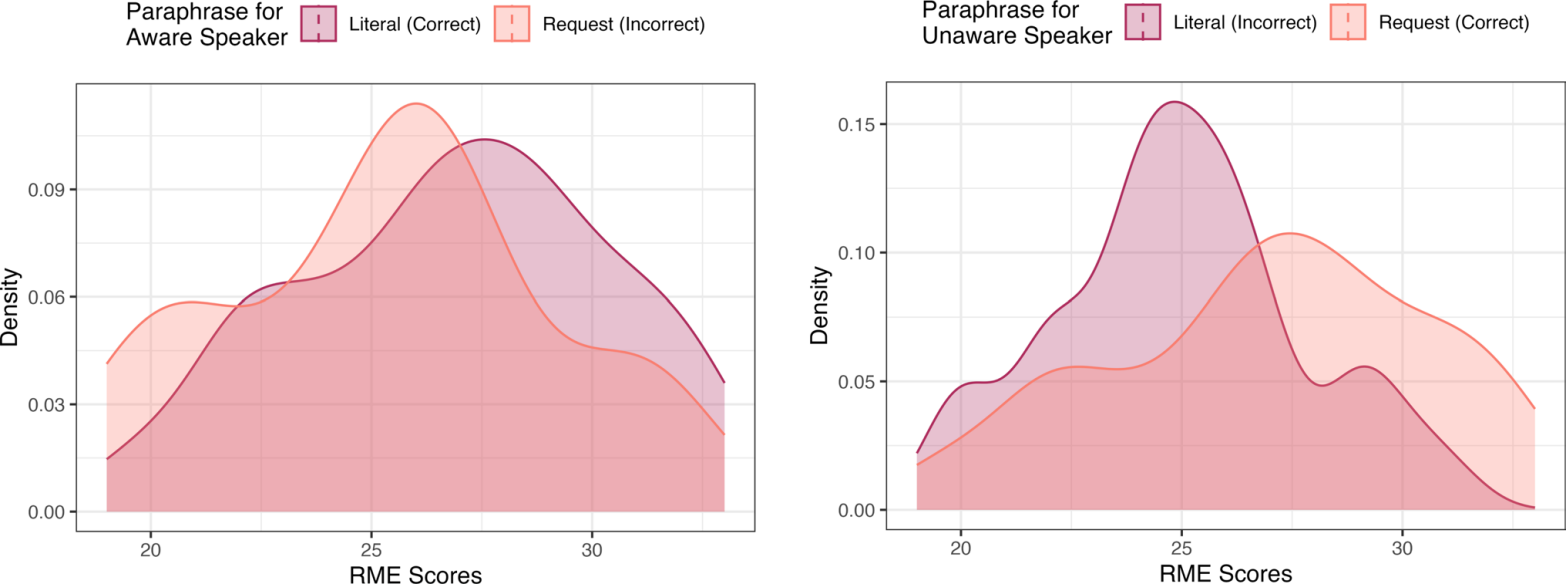
Left: Kernal density estimates of distribution of RME scores for (1) participants who interpreted aware speakers as making literal comments/questions; (2) participants who interpreted aware speakers as making requests; Right: Kernal density estimates of distribution of RME scores for (1) participants who interpreted unaware speakers as making literal comments/questions; (2) participants who interpreted unaware speakers as making requests

To explore the significant interaction effect for Condition and RME scores, we ran a post-hoc analysis, where we flipped the model, making RME scores txhe outcome variable, and Condition and Interpretation the predictor variables, again with Item included as a random effect. We then calculated Tukey HSD tests using the emmeans package in R-Studio (Lenth, 2023) to compare RME scores for participants who selected one or the other paraphrase choice for one or the other condition. Results revealed significant differences in RME scores for two comparisons, and marginal differences for the other two comparisons. First, we will examine comparisons for the two conditions. In the Speaker Aware condition, individuals who “correctly” identified utterances as literal comments/questions (95% *CI* for RME scores = [26.8, 29.8]) had marginally higher RME scores than participants who “incorrectly” identified utterances as request (95% *CI* for RME scores = [25.3, 28.3], *t* = 2.00, *p* = 0.190). In the Speaker Unaware condition, individuals who correctly identified utterances as requests (95% *CI* for RME scores = [23.6, 26.3]) had significantly higher RME scores than participants who incorrectly interpreted utterances literally (95% *CI* for RME scores = [21.6, 23.4], *t* = − 2.96, *p* = 0.017). See Fig. 1.

**Fig. 2:**
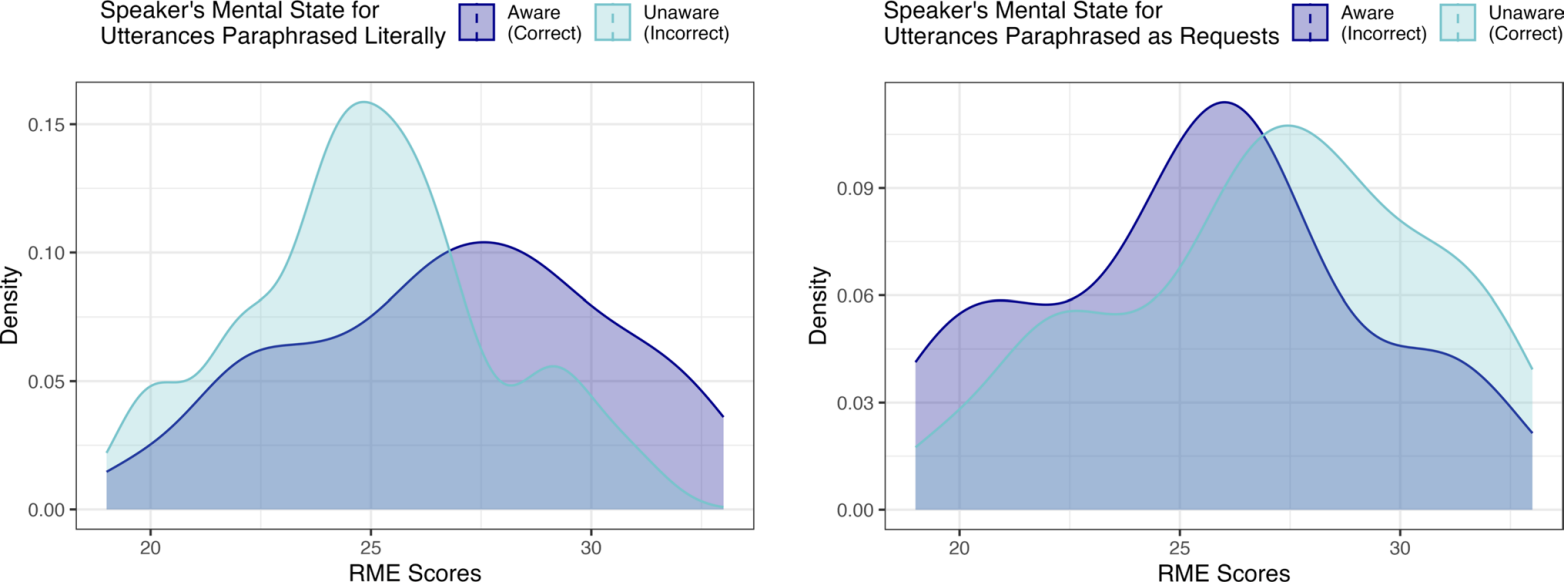
Left: Kernal density estimates of distribution of RME scores for (1) participants who attributed literal paraphrases to aware speakers; (2) participants who attributed request paraphrases to aware speakers; Right: Kernal density estimates of distribution of RME scores for (1) participants who attributed literal paraphrases to unaware speakers; (2) participants who attributed request paraphrases to unaware speakers

Second, we will examine comparisons for the two responses. Individuals who correctly ascribed literal interpretations in the Speaker Aware condition (95% *CI* for RME scores = [26.8, 29.8]) had significantly higher RME scores than individuals who incorrectly ascribed literal interpretations in the Speaker Unaware condition (95% *CI* for RME scores = [21.6, 24.3], *t* = 2.79, *p* = 0.028). Mean RME scores for individuals who incorrectly ascribed request interpretations in the Speaker Aware condition (95% *CI* for RME scores = [25.4, 28.3]) were slightly (non-significantly) lower than those who correctly ascribed request interpretations in the Speaker Unaware condition (95% *CI* for RME scores = [23.6, 26.3], *t* = -2.16, *p* = 0.136). See Fig. 2.

Then, we examined the influence of each EF skill, on its own (in addition to Condition). Beginning with Working Memory, the model including N-Back scores and Condition as fixed effects, along with the interaction between them, marginally improved model fit compared to the original, reduced model [χ^2^(2) = 5.25, *p* = 0.07]. Independent-samples t-tests revealed that significant differences for N-Back scores were only present in the Speaker Unaware condition. Participants who correctly recognized that utterances produced by *unaware speakers* were intended as requests (95% *CI* for RME scores = [21.1, 24.1]) had marginally significantly higher N-Back scores than participants who chose literal interpretations (95% *CI* for RME scores = [19.5, 22.4], *t* = -2.74, *p* = 0.03). To determine the relative influence of Working Memory, we compared models containing RME and N-Back scores to models with just N-Back scores or RME scores. The model including RME and N-Back scores, significantly improve model fit compared to the model with just N-Back [χ^2^(2) = 9.33, *p* = 0.009], but the model including N-Back and RME scores, did not significantly improve model fit compared to the model with just RME [χ^2^(2) = 2.42, *p* = 0.30]. This shows that the RME scores had a greater influence on participants’ performance on the indirect request task than Working Memory.

Next, a model with Inhibition (measured using the Flanker task) and Condition as fixed effects, along with the interaction between both, showed a non-significant improvement in model fit [χ^2^(2) = 3.52, *p* = 0.17]. The model with Attention Shifting (measured using the WCST) and Condition as fixed effects, along with the interaction between both, showed a similar non-significant improvement of model fit compared to the reduced model [χ^2^(2) = 2.20, *p* = 0.33]. Finally, to determine whether a combination of Inhibition and RME or Attention Shifting and RME accounted for a significant portion of the variance on indirect request interpretation, we created two models, where scores on each EF subskill—and all interactions—were added to the as-yet best fitting model: One with RME scores and Condition as fixed effects, along with the interaction between them. Neither of the comparisons showed a significant improvement of model fit [Flanker: χ^2^(2) = 1.22, *p* = 0.54; WCST: χ^2^(2) = 2.19, *p* = 0.33].

### Response time

**Fig. 3:**
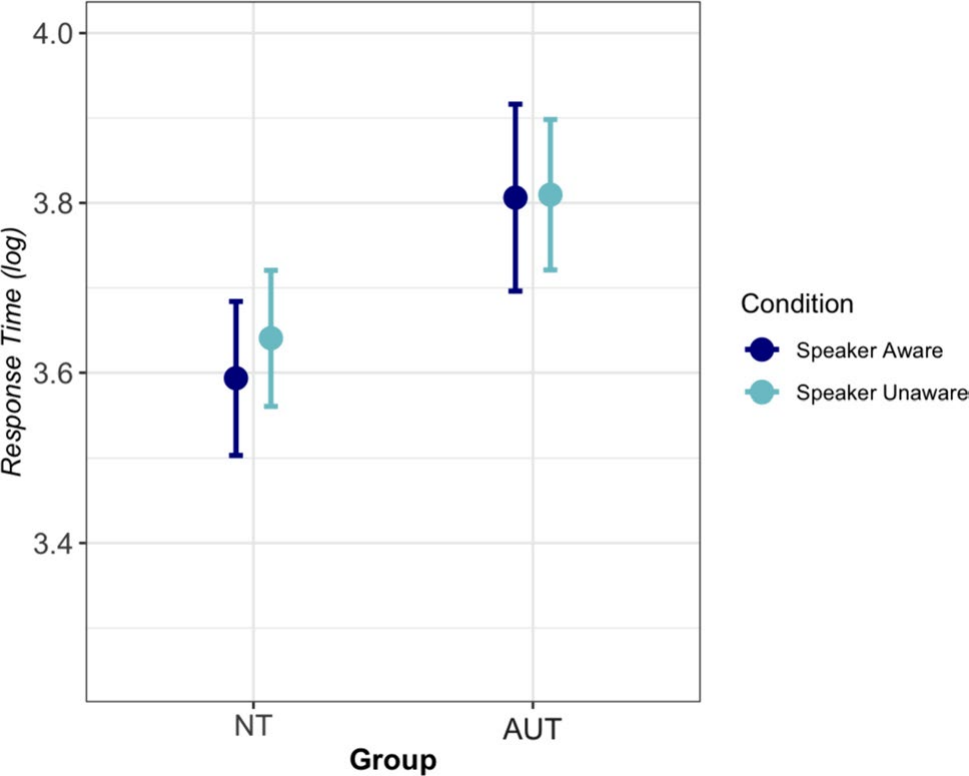
Average response time (log) from onset of passage presentation to response for the NT and AUT groups in each condition with standard error

**Fig. 4:**
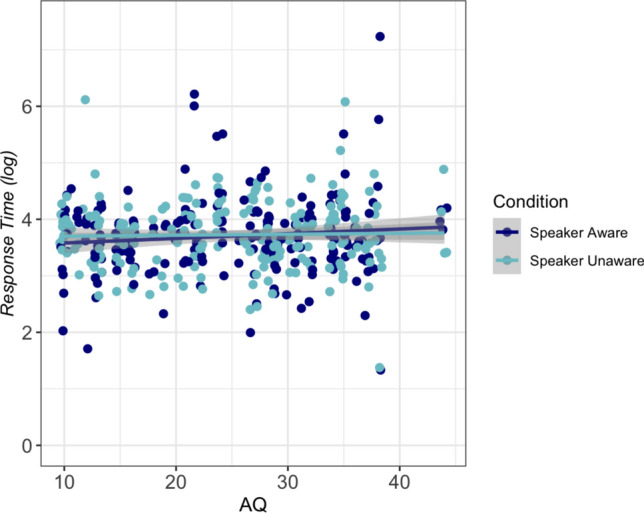
Scatter and line plot showing relationship between response time (log) from onset of passage to response by AQ scores and Condition

Although this study was not designed as a processing study, given the lack of a group effect in the interpretation data, we conducted a post-hoc analysis of the response time (RT) data. Since we did not originally intend to perform a RT analysis, the length of the passages, while similar across conditions, was not controlled for, and therefore is included as a factor in statistical analyses that involve the condition variable. Before analysis, RT data was log-transformed to increase normality and deal with outliers, a treatment that has been recommended for RT outliers over trimming or winsorizing based on standard deviation cutoffs (Nicklin and Plonsky 2020). The AUT group had longer response times than the NT group on average; however, this difference was not statistically significant. Models with fixed effects based on group did not statistically improve a null model with random effects of participant and item alone, regardless of whether a binary group variable [χ^2^(1) = 2.59, *p* = 0.11] (Fig. 3) or AQ [χ^2^(1) = 0.57, *p* = 0.45] (Fig. 4) was used to operationalized group status. A model that added a fixed effect of Condition (Speaker Aware vs. Speaker Unaware) also did not improve a model with a fixed effect of passage Length and random effects of participant and item [χ^2^(1) = 0.01, *p* = 0.91].

Given a few extreme outliers, we once again ran the analysis, this time winsoring response times with z-scores greater than ± 2 SD (based on participant means) to deal with outliers before using log transformation to normalize the positive skew (again, following Nicklin and Plonsky 2020 who advise winsoring over trimming). The outliers were replaced with scores equivalent to the participant mean plus 2 SD. Participant means were chosen over group/condition means since, as mentioned above, the experiment was not designed to collect response time data. As such, participants were not instructed to read the passages as quickly as possible, nor were explicit breaks built-in, potentially meaning that long outliers (e.g., 1400 s) were due to participants getting up from the computer in the middle of a random item for a break, rather than being influenced by a particular experimental condition.

The resulting data set was used for statistical modeling, with the results similar to those reported above. Fixed effects based on group did not statistically improve the null model with random effects of participant and item alone, whether operationalized as a binary [χ^2^(1) = 2.56, *p* = 0.11] or continuous AQ variable [χ^2^(1) = 0.56, *p* = 0.46]. A model that added a fixed effect of Condition once again did not improve a model with a fixed effect of passage Length and random effects of participant and item [χ^2^(1) = 0.01, *p* = 0.91].

## Discussion

This study is the first to examine whether autistic adults can use discourse context to interpret whether a “hint”—a non-conventionalized request form that does not contain the form and/or semantic components of the underlying imperative—is intended literally or not. Two research questions were addressed: (1) How do autistic adults compare to NT adults in using discourse context to interpret an utterance’s intention as either a request or a comment/question? (2) What cognitive mechanisms correlate with performance on indirect request interpretation, and are these different for participants in each group?

To address Question 1, we assessed autistic and NT adults on their ability to determine whether an utterance is an indirect request or a literal comment/question, based on discourse context. Results indicated that autistic adults performed similarly to NT adults on the indirect request task. Additionally, one’s level of autistic features, as measured by the AQ, had no correlation with performance. While this finding is consistent with previous research showing that hint comprehension is intact for autistic children (Kissine et al. 2015; MacKay and Shaw 2004; Marocchini et al. 2021), it contradicts our initial hypothesis. We hypothesized that autistic adults would provide less correct responses on the indirect request task based on the WCC theory as well as our assumption that they would score lower on ToM tasks, which have been shown to correlate with NT adults’ indirect request performance on an equivalent task (Trott and Bergen 2018; 2020). For the current study, participants were required to not only consider the discourse context but also incorporate the speakers’ mental states in order to determine intentionality. The fact that autistic participants were successful at this task suggests that they can incorporate both elements into their interpretation of an ambiguous utterance. Consequently, our findings do not support the theory of WCC (Happé and Frith 2006); autistic adults are just as skilled as NT adults at using discourse context to guide their interpretations of hints.

To address Question 2, we first investigated the link between ToM and hint interpretation. Participants completed two ToM tasks: the RME and a false belief task. We found that scores from the RME predicted performance on the indirect request task. Specifically, participants with lower RME scores provided more incorrect responses in both the Speaker Aware and Speaker Unaware conditions. This finding supports research from both NT adults (Trott and Bergen 2018; 2020; van Ackeren et al. 2012) and adults with other disorders affecting pragmatics, such as RHD (Champagne-Lavau and Joanette 2009), that ToM is involved during hint interpretation. Complicating this interpretation, however, is the fact that our second ToM measure, the false belief task, was not predictive of individuals’ performance on the indirect request task.

The decision to include two ToM tasks was based on the growing idea that the notion of “ToM” itself involves numerous, multifaceted subcomponents of human cognition, which may or may not actually assemble into one discrete process that can be measured by one overall ToM test (Quesque and Rossetti 2020). Further, tests that purportedly measure ToM often rely on unrelated areas of cognition and language. Beginning with false belief tasks, evidence suggests that verbal abilities strongly correlate with performance, and that as autistic individuals reach adolescence, they tend perform similarly on such tasks to their NT peers (Happé, 1995; Scheeren et al. 2013), perhaps due to comparable language abilities. While we did find significant differences between the groups on the false belief task, the majority of participants from both groups showed close-to-ceiling level performance, with 21 out of 26 participants in the AUT group, and 25 out of 26 participants in the NT group, earning perfect scores on the task.

This correlation between language abilities and such tasks motivated our inclusion of the RME, a ToM task that should avoid correlations with reading comprehension. However, the RME is not without its own problems. Several studies have indicated that performance on the RME is more related to factors such as intelligence, vocabulary, and emotional recognition, rather than mentalizing (Baker et al. 2014; Oakley et al. 2016; Olderbak et al. 2015). In fact, some authors purport that tests requiring participants to identify emotional expressions from pictures do not measure ToM at all, arguing that true ToM tasks should not only test whether participants can determine someone’s mental state, but also distinguish their own mental state from that person’s (Oakley et al. 2016; Quesque and Rossetti 2020). Indeed, when we examined the relationship between participants’ performance on the RME and the false belief task, in the current study, we found a very weak *negative* correlation (*r* = − 0.034, *p* = 0.81).

In addition to ToM, we examined the effect of EF on indirect request interpretation. For EF, we initially hypothesized that autistic participants would score lower than NT participants, and that these scores would be predictive for how individuals performed on the indirect request task. It was found that NT participants only showed an advantage on the attention shifting task. The groups showed similar inhibition skills, while the AUT group had significantly greater working memory skills. This confirms research from a few other studies, which have found that autistic college students score within normal ranges on EF measures (Brady et al. 2017; Toyb et al. 2014). Regarding the predictive effects of EF, we found that only working memory, as measured through a n-back task, predicted a significant portion of variance on the indirect request task. However, working memory was less predictive of variance than the RME scores, combining n-back and RME scores did not enhance the predictive value of a model, suggesting that of all the cognitive skills tested in the current study, emotion recognition was best at predicting utterance interpretation.

While findings for Question 2 contradicted many of our initial hypotheses, it should be noted that these findings indicate that autistic and NT adults use similar cognitive mechanisms when utilizing discourse context to interpret whether or not a speaker intends to be literal or not. This is significant given that previous research has suggested that autistic children do not use the same cognitive mechanisms as the NT peers during indirect request interpretation (Kissine et al. 2015; Marocchini et al. 2021). It is possible that as autistic individuals age, and their ToM skills become more developed (see Gernsbacher and Yergeau 2019 for a review), they are able to rely more on mentalizing (or at least certain mentalizing subskills, like emotion recognition) to interpret hints.

### Linguistic vs. social pragmatics

The fact that indirect request comprehension is intact in autistic populations lies in stark contrast to the consistent evidence that autistic individuals show differences in their understanding of other types of non-literal language, such as irony (e.g., Deliens et al. 2018; Happé, 1993). Andrés-Roqueta and Katsos (2017; 2020) have suggested a dichotomy between linguistic pragmatics and social pragmatics to provide insight into why autistic individuals are proficient in certain pragmatic tasks and not others. In this framework, linguistic pragmatics encompasses pragmatics tasks that only require linguistic skills and basic knowledge of pragmatic norms for successful comprehension (e.g., scalar implicatures). Conversely, social pragmatics encompasses tasks that demand not only linguistic and pragmatic understanding, but also the engagement of ToM (e.g., irony). Researchers have suggested that the intact comprehension of indirect requests, in contrast to poorer performance on other forms of non-literal language, like irony, in autistic populations supports the existence of these two types of pragmatics (Andrés-Roqueta and Katsos 2020; Deliens et al. 2018; Marocchini et al. 2021). For example, Deliens and colleagues (2018) argued that the request forms utilized in their study were an example of linguistic pragmatics, given that listeners can rely on the conventionalized structural frame (e.g., “Can you…?”) to interpret speaker meaning. While these conventionalized forms may fall within the category of linguistic pragmatics, this may not be the case for hints.

Given that hints, by definition, are “not on the surface directives” (Ervin-Tripp 1976, p. 42), listeners must utilize context and inferencing to reach the directive interpretation. Indeed, research from both clinical (Champagne-Lavau and Joanette 2009) and non-clinical (Trott and Bergen 2018; 2020) adult populations have shown ToM skills to be related to hint interpretation. Additionally, van Ackeren and colleagues (2012) found brain regions associated with mentalizing were activated during indirect request interpretation in NT adults. As such, results from these studies suggest that hints should be classified as social pragmatics. It is therefore unexpected that the autistic participants did not differ from the NT group in this study.

In order to explain this result, it is necessary to go beyond the linguistic vs. social pragmatics dichotomy. Our results indicate that while understanding false beliefs may not be involved in the interpretation of hints, other mentalizing subskills, like emotional recognition, may be implicated. Therefore, we contend that the difference between indirect requests and irony lies not in the demand for mentalizing, overall, as others have suggested (Andrés-Roqueta and Katsos 2020; Deliens et al. 2018; Marocchini et al. 2021), but in the type of mental state decoding required for each. In our indirect request task, participants only needed to decode the speaker’s level of awareness to be successful. We argue that this type of mental state decoding is more straightforward than the mental state decoding needed to interpret irony. Interpreting irony requires the listener to attribute a mental state to the speaker that is often the *opposite* of what is said, and usually involves decoding para- and extra-linguistic cues, like intonation and facial expressions, respectively (Attardo et al. 2003). Hence, irony demands a more multi-layered analysis in contrast to the relatively simpler decoding involved in indirect request comprehension.

### Limitations and future research

It should be noted that successful performance on the indirect request task used in the current study may not necessarily implicate real-life performance. The current experiment was untimed, allowing participants to take as long as they needed to interpret a given utterance’s intention. This aspect of the current study could explain why there was no effect of EF on individuals’ performances on the indirect request task. In real-life conversations that require quick processing and the inhibition of distracting stimuli, EF may have a more significant effect on indirect request interpretation.

Additionally, participants were given a binary choice for their paraphrase, while in real, interactional situations, people have unlimited possibilities for their interpretation of an utterance. Further, specific aspects of the binary choices offered to participants makes it difficult to determine whether participants truly recognized the final utterance. As in Trott and Bergen (2018; 2020), literal selections (e.g., I’m really cold) always included additional information that speakers would have no way of knowing in the speaker unaware condition (e.g., “I’m really cold; it’s too bad the heater is broken”.). Thus, if participants were able to use story context to accurately understand the speaker’s background knowledge, they could eliminate the literal interpretation without necessarily recognizing that the speaker intended to request. To address this limitation, a better experimental design might involve offering interpretation options that leave out facts that are impossible to know in the unaware condition (e.g., “I am really cold”). Because amending the literal option this way would make both options true in the speaker unaware condition, the paradigm could be altered so that participants are asked to select all that apply. In the aware condition, we would expect participants to only pick “I am really cold,” while in the speaker unaware condition, if they recognize “I’m really cold” is an indirect request, they could still select “I am really cold” (since that may technically be true), but they would also need to pick “Can you turn on the heater?” to show they recognize the speaker’s likely intention to request.

Another limitation of the current study is that we did not include a measure of online processing. Although we used response times as a gross reflection of processing, the experiment was not designed to collect RT data. Participants were not encouraged to answer questions as quickly as possible, nor were their sessions monitored in any way. Thus, long response times might be attributed to participants taking breaks and/or disengaging from the task, rather than reflecting specific experimental conditions. Consequently, RT data should be interpreted with caution.

Based on these limitations, future research should allow participants to either select multiple paraphrases or provide their own paraphrases, should use auditory stimuli (with inherent time constraints) or should otherwise limit the time allowed to respond, and should measure online processing, using measures like eye-tracking or self-paced reading. Furthermore, it is crucial to consider the heterogeneity within the autistic population, given that we only tested autistic college students. Conducting future studies with autistic adults at different educational levels would provide a more comprehensive understanding of indirect request comprehension in this population.

Finally, we recommend further research to explore how autistic children interpret hints, specifically assessing how children utilize context to determine whether or not an utterance is intended to be an indirect request. Given that our findings suggest that (at least some) ToM subskills are involved in indirect request interpretation for all participants, and because previous research fairly consistently finds differences in scores on ToM tasks for autistic children (e.g., Baron-Cohen 2000), it is possible that autistic children would perform differently on this task than NT children and/or that cognitive mechanisms aside from ToM (e.g., EF) would contribute to their understanding.

## Conclusion

This study investigated how autistic college students comprehend hints, which are indirect requests that do not contain the form and/or semantic components of the underlying imperative. Results revealed that hint comprehension is similar for autistic and NT college students, which challenged our hypothesis that autistic adults would perform less accurately due to assumed lower ToM abilities. Another unexpected finding was that only certain mentalizing skills, specifically emotion recognition, predicted how participants utilized context to determine if an utterance was intended as a hint. These results have implications for the linguistic- versus social-pragmatics framework (Andrés-Roqueta and Katsos 2017; 2020), since hint interpretation should arguably be categorized as social-pragmatics, as it involves both linguistic knowledge and mentalizing, yet autistic adults seem in to interpret hints well. Based on our findings and those from previous work, we suggest that successful hint comprehension differs from the understanding of other types of non-literal language, like irony. It seems that the simple, binary split between linguistic and social pragmatics is insufficient for predicting autistic individuals’ comprehension patterns; a more comprehensive model may need to accommodate how the interface between (social) pragmatics and para-/extra-linguistic information affects interpretation.

## Speaker aware

You and your friend Jonathan are taking a road trip. You began in California, and are now passing through Michigan. It’s almost winter, so it’s very cold outside—especially for Southern California dwellers like you and Jonathan. You see that you’re almost out of gas, so you stop at a gas station in a small town.

You fill up the tank, and then the two of you go inside the gas station to buy some water and snacks. When you return to the car and start up the engine, you and Jonathan notice with some dismay a blinking light, which indicates that the car’s heating system is broken. You both bundle up.

As you leave the station, Jonathan shivers in his seat. He turns to you and says, “Man, it’s really cold in here”.

## Speaker unaware

You and your friend Jonathan are taking a road trip. You began in California, and are now passing through Michigan. It’s almost winter, so it’s very cold outside—especially for Southern California dwellers like you and Jonathan. You see that you’re almost out of gas, so you stop at a gas station in a small town.

While you fill up the tank, Jonathan goes inside to buy some water and snacks. As you’re checking the meter, you notice with some dismay a blinking light, which indicates that the car’s heating system is broken. You finish filling up the gas and wait for Jonathan.

Jonathan returns with some snacks, and you both set off. As you leave the station, he shivers in his seat. He turns to you and says, “Man, it’s really cold in here”.

## Paraphrase options

Could you turn on the heater?

I’m really cold; it’s too bad the heater is broken.

## Question 1

Anthony has just returned home from the gym. He puts his cell phone and wireless headphones on the kitchen table and goes upstairs to shower. While Anthony is showering, his roommate, Sean, goes into the kitchen to make some lunch. While Sean is making his lunch, he hears some faint music coming from Anthony's headphones. Apparently, Anthony had forgotten to turn off his music after returning home from the gym. Sean can't turn off the music because he doesn't know Anthony's phone's passcode, and even after trying to turn down the phone's volume, he can still hear the music. Sean starts to get annoyed by the music, so he puts the phone and headphones in Anthony's gym bag.

After Anthony is showered and dressed, he remembers he needs to text a friend.

Where does he look for his phone?

- Table
- Gym bag
- Shelf
- Chair

## Question 2

Rachel has just finished baking cookies for her and her kids. She always makes two kinds of cookies: Peanut butter cookies for herself and chocolate chip cookies for her kids, who both have a mild peanut allergy. Because of her kids’ allergies, it is important that she keeps her cookies separate from theirs and that it’s clear to everyone which cookies are which. To do that, she always puts the chocolate chip cookies in a blue jar on the dining-room table and the peanut butter cookies in a red jar on the kitchen counter. After she puts the cookies in their respective places, she goes out to run some errands and to pick up her kids from school. While she’s away, a cleaning company is scheduled to come clean her house. The cleaners move both cookie jars so they can clean the table and the counter, and when they put the jars back, they mix up which one goes where: They put the red jar on the table and the blue jar on the counter.

When Rachel comes in with her kids after school, she tells them there is a jar of fresh-baked cookies on the table for them.

Which jar does Rachel think is on the table?

- Red jar of peanut butter cookies
- Blue jar of chocolate chip cookies
- Red jar of chocolate chip cookies
- Blue jar of peanut butter cookies

## Question 3

Henry’s birthday is this weekend. On Wednesday, he sees an Instagram post announcing that his favorite band has a last-minute performance planned at a nearby venue on Friday. He forwards the post to his girlfriend, Jada, and comments that going to see the band would be a perfect birthday celebration. Later that day, Jada tells Henry that she tried to get tickets, but they were already sold out. She suggests going to the movies instead. On Friday morning while Jada is taking a shower, an event alarm goes off on her phone. Henry glances at it and sees that it’s a reminder for the concert that evening. He realizes that Jada was able to get tickets to the concert, after all. Henry does not tell Jada about his discovery.

Later that evening, Jada tells Henry it’s time to go to the movies. She says that she’ll drive them to the movie theater.

Where does Henry think he’s going?

- Restaurant
- Museum
- Movies
- Concert

Where does **Jada** think Henry thinks he’s going?

- Restaurant
- Museum
- Movies
- Concert

## Footnotes

1 We have changed “imbedded”—which is Ervin-Tripp’s original spelling of the word—to “embedded” for the rest of the paper.

2 For this paper, we intentionally use identity-first language (i.e., autistic person) to be consistent with the preference of the autism community (Brown 2011).

## Declarations

### Human and/or animal participation

The article does not contain any studies involving animals performed by any of the authors. All procedures performed in studies involving human participants were in accordance with the ethical standards of the institutional and/or national research committee and with the 1964 Helsinki Declaration and its later amendments or comparable ethical standards.

## References

[CR71] Ackerman BP (1978) Children's understanding of speech acts in unconventional directive frames. Child Development 49(2):311–318. 10.2307/1128692

[CR1] Andrés-Roqueta C, Katsos N (2017) The contribution of grammar, vocabulary and theory of mind in pragmatic language competence in children with autistic spectrum disorders. Front Psychol 8:996. 10.3389/fpsyg.2017.00996 28663734 10.3389/fpsyg.2017.00996 PMC5471327

[CR2] Andrés-Roqueta C, Katsos N (2020) A distinction between linguistic and social pragmatics helps the precise characterization of pragmatic challenges in children with autism spectrum disorders and developmental language disorder. J Speech Lang Hear Res JSLHR 63(5):1494–1508. 10.1044/2020_JSLHR-19-00263 32379523 10.1044/2020_JSLHR-19-00263

[CR3] Attardo S, Eisterhold J, Hay J, Poggi I (2003) Multimodal markers of irony and sarcasm. Humor Int J Humor Res 16(2):243–260. 10.1515/humr.2003.012

[CR4] Babelot G, Marcos H (1999) Comprehension of directives in young children: influence of social situation and linguistic form. First Lang 19(56):165–186. 10.1177/014272379901905602

[CR5] Baker CA, Peterson E, Pulos S, Kirkland RA (2014) Eyes and IQ: a meta-analysis of the relationship between intelligence and “Reading the Mind in the Eyes.” Intelligence 44:78–92. 10.1016/j.intell.2014.03.001

[CR6] Baron-Cohen S (2000) Theory of mind and autism: a review. Int Rev Res Mental Retard 23:169–184. 10.1016/S0074-7750(00)80010-5

[CR7] Baron-Cohen S, Leslie AM, Frith U (1985) Does the autistic child have a “theory of mind”? Cognition 21(1):37–46. 10.1016/0010-0277(85)90022-8 2934210 10.1016/0010-0277(85)90022-8

[CR8] Baron-Cohen S, Wheelwright S, Hill J, Raste Y, Plumb I (2001a) The “Reading the Mind in the Eyes” test revised version: a study with normal adults, and adults with asperger syndrome or high-functioning autism. J Child Psychol Psychiatr Allied Discipl 42(2):241–251. 10.1111/1469-7610.00715 11280420

[CR9] Baron-Cohen S, Wheelwright S, Skinner R, Martin J, Clubley E (2001b) The autism-spectrum quotient (AQ): evidence from Asperger syndrome/high-functioning autism, males and females, scientists and mathematicians. J Autism Dev Disord 31(1):5–17. 10.1023/a:1005653411471 11439754 10.1023/a:1005653411471

[CR10] Bates D, Mächler M, Bolker B, Walker S (2015) Fitting linear mixed-effects models using lme4. J Stat Softw 67(1):1–48. 10.18637/jss.v067.i01

[CR11] Berg EA (1948) A simple objective technique for measuring flexibility in thinking. J General Psychol 39(1):15–22. 10.1080/00221309.1948.9918159 10.1080/00221309.1948.9918159 18889466

[CR12] Bernicot J, Legros S (1987) Direct and indirect directives: What do young children understand? J Exp Child Psychol 43(3):346–358. 10.1016/0022-0965(87)90012-9

[CR13] Bernicot J, Laval V, Chaminaud S (2007) Nonliteral language forms in children: In what order are they acquired in pragmatics and metapragmatics? J Pragmat 39:2115–2132. 10.1016/j.pragma.2007.05.009

[CR14] Brady DI, Saklofske DH, Schwean VL, Montgomery JM, Thorne KJ, McCrimmon AW (2017) Executive functions in young adults with autism spectrum disorder. Focus Autism Other Develop Disabil 32(1):31–43. 10.1177/1088357615609306

[CR69] Brown P, Levinson SC (1987) Politeness: some universals in language usage. Cambridge University Press, Cambridge. 10.1017/CBO9780511813085

[CR15] Brown VL, Wiederholt JL, Hammill DD (2009) Test of reading comprehension-fourth edition (TORC-4). Austin, TX: Pro-Ed

[CR16] Brown L (2011) Identity-first language. Autistic self advocacy network. http://autisticadvocacy.org/about-asan/identity-first-language/

[CR17] Bucciarelli M, Colle L, Bara BG (2003) How children comprehend speech acts and communicative gestures. J Pragmat 35(2):207–241. 10.1016/S0378-2166(02)00099-1

[CR20] Champagne-Lavau M, Joanette Y (2009) Pragmatics, theory of mind and executive functions after a right-hemisphere lesion: different patterns of deficits. J Neurolinguistics 22(5):413–426. 10.1016/j.jneuroling.2009.02.002

[CR21] de Leeuw JR (2015) jsPsych: a JavaScript library for creating behavioral experiments in a web browser. Behav Res Methods 47(1):1–12. 10.3758/s13428-014-0458-y 24683129 10.3758/s13428-014-0458-y

[CR22] De Mulder H (2015) Developing communicative competence: a longitudinal study of the acquisition of mental state terms and indirect requests. J Child Lang 42(5):969–1005. 10.1017/S0305000914000543 25262752 10.1017/S0305000914000543

[CR23] Deliens G, Papastamou F, Ruytenbeek N, Geelhand P, Kissine M (2018) Selective pragmatic impairment in autism spectrum disorder: indirect requests versus irony. J Autism Dev Disord 48:2938–2952. 10.1007/s10803-018-3561-6 29633109 10.1007/s10803-018-3561-6

[CR24] Dodell-Feder D, Lincoln SH, Coulson JP, Hooker CI (2013) Using fiction to assess mental state understanding: a new task for assessing theory of mind in adults. PLoS ONE 8(11):e81279. 10.1371/journal.pone.0081279 24244736 10.1371/journal.pone.0081279 PMC3820595

[CR25] Elrod MM (1987) Children’s understanding of indirect requests. J Genet Psychol Res Theory Human Develop 148(1):63–70. 10.1080/00221325.1987.9914537

[CR26] Eriksen BA, Eriksen CW (1974) Effects of noise letters upon the identification of a target letter in a nonsearch task. Percept Psychophys 16(1):143–149. 10.3758/BF03203267

[CR27] Ervin-Tripp (1976) Is Sybil there? The structure of some American English directives. Lang Soc 5(1):25–66. 10.1017/S0047404500006849

[CR28] Ervin-Tripp S (1977) Wait for me, Roller Skate! In Ervin-Tripp S, Mitchell-Kernan C (Eds) Child Discourse (pp. 165–188). New York: Academic Press. 10.1016/B978-0-12-241950-8.50015-0

[CR29] Gernsbacher MA, Yergaeu M (2019) Empirical failures of the claim that autistic people lack a theory of mind. Arch Sci Psychol 7(1):102–118. 10.1037/arc0000067 31938672 10.1037/arc0000067 PMC6959478

[CR30] Gibbs RW (1986) What makes some indirect speech acts conventional? J Mem Lang 25(2):181–196. 10.1016/0749-596X(86)90028-8

[CR31] Happé FGE (1993) Communicative competence and theory of mind in autism. A test of relevance theory. Cognition 48(2):101–119. 10.1016/0010-0277(93)90026-R 8243028 10.1016/0010-0277(93)90026-r

[CR32] Happé FG (1994) An advanced test of theory of mind: understanding of story characters’ thoughts and feelings by able autistic, mentally handicapped, and normal children and adults. J Autism Dev Disord 24(2):129–154. 10.1007/BF02172093 8040158 10.1007/BF02172093

[CR33] Happé FG (1995) The role of age and verbal ability in the theory of mind task performance of subjects with autism. Child Dev 66(3):843–855 7789204

[CR34] Happé F, Frith U (2006) The weak coherence account: Detail-focused cognitive style in autism spectrum disorders. J Autism Dev Disord 36(1):5–25. 10.1007/s10803-005-0039-0 16450045 10.1007/s10803-005-0039-0

[CR35] Howlin P, Magiati I (2017) Autism spectrum disorder: Outcomes in adulthood. Curr Opin Psychiatry 30(2):69–76. 10.1097/YCO.0000000000000308 28067726 10.1097/YCO.0000000000000308

[CR36] Jeffreys H (1939) Theory of probability, 1st. Oxford University

[CR37] Jolliffe T, Baron-Cohen S (1999) The strange stories test: a replication with high-functioning adults with autism or asperger syndrome. J Autism Dev Disord 29:395–406. 10.1023/A:1023082928366 10587886 10.1023/a:1023082928366

[CR39] Kissine M, De Brabanter P, Leybaert J (2012) Compliance with requests by children with autism: the impact of sentence type. Autism 16(5):523–531. 10.1177/1362361311406296 22399448 10.1177/1362361311406296

[CR40] Kissine M, Cano-Chervel J, Carlier S, De Brabanter P, Ducenne L, Pairon M-C, Deconinck N, Delvenne V, Leybaert J (2015) Children with autism understand indirect speech acts: Evidence from a semi-structured act-out task. PLoS ONE. 10.1371/journal.pone.0142191 26551648 10.1371/journal.pone.0142191 PMC4638355

[CR70] Lakoff R (1973) The logic of politeness, or minding your P’s and Q’s. Papers from the 9th Regional Meeting of the Chicago Linguistic Society. Chicago Linguistics Society 9:292–305

[CR41] Ledbetter PJ, Dent CH (1988) Young children’s sensitivity to direct and indirect request structure. First Lang 8(24):227–246. 10.1177/014272378800802402

[CR42] Leonard LB, Wilcox MJ, Fulmer KC, Davis GA (1978) Understanding indirect requests: an investigation of children’s comprehension of pragmatic meanings. J Speech Hear Res 21(3):528–537. 10.1044/jshr.2103.528 713521 10.1044/jshr.2103.528

[CR43] Lewis LF (2017) A mixed methods study of barriers to formal diagnosis of autism spectrum disorder in adults. J Autism Dev Disord 47(8):2410–2424. 10.1007/s10803-017-3168-3 28516422 10.1007/s10803-017-3168-3

[CR44] Liebling CR (1988) Means to an end: children’s knowledge of directives during the elementary school years. Discourse Process 11:79–99. 10.1080/01638538809544692

[CR45] MacKay G, Shaw A (2004) A comparative study of figurative language in children with autistic spectrum disorders. Child Lang Teach Therapy 20(1):13–32. 10.1191/0265659004ct261oa

[CR46] Marocchini E, Di Paola S, Mazzaggio G, Domaneschi F (2021) Understanding indirect requests for information in high-functioning autism. Cogn Process 23:129–153. 10.1007/s10339-021-01056-z 34487273 10.1007/s10339-021-01056-z PMC8831260

[CR47] Milton DEM (2012) On the ontological status of autism: the ‘double empathy problem.’ Disabil Soc 27(6):883–887. 10.1080/09687599.2012.710008

[CR48] Miyake A, Friedman NP, Emerson MJ, Witzki AH, Howerter A, Wager TD (2000) The unity and diversity of executive functions and their contributions to complex “frontal lobe” tasks: a latent variable analysis. Cogn Psychol 41(1):49–100. 10.1006/cogp.1999.0734 10945922 10.1006/cogp.1999.0734

[CR49] Nicklin C, Plonsky L (2020) Outliers in L2 research in applied linguistics: a synthesis and data re-analysis. Annu Rev Appl Ling 40:26–55. 10.1017/S0267190520000057

[CR50] Oakley BFM, Brewer R, Bird G, Catmur C (2016) Theory of mind is not theory of emotion: a cautionary note on the reading the mind in the eyes test. J Abnorm Psychol 125(6):818–823. 10.1037/abn0000182 27505409 10.1037/abn0000182 PMC4976760

[CR51] Olderbak S, Wilhelm O, Olaru G, Geiger M, Brenneman MW, Roberts RD (2015) A psychometric analysis of the reading the mind in the eyes test: toward a brief form for research and applied settings. Front Psychol 6:1503. 10.3389/fpsyg.2015.01503 26500578 10.3389/fpsyg.2015.01503 PMC4593947

[CR52] Ozonoff S, Miller JN (1996) An exploration of right-hemisphere contributions to the pragmatic impairments of autism. Brain Lang 52(3):411–434. 10.1006/brln.1996.0022 8653388 10.1006/brln.1996.0022

[CR53] Paul R, Cohen DJ (1985) Comprehension of indirect requests in adults with autistic disorders and mental retardation. J Speech Hear Res 28(4):475–479. 10.1044/jshr.2804.475 4087881 10.1044/jshr.2804.475

[CR72] Quesque F, Rossetti Y (2020) What do theory-of-mind tasks actually measure? Theory and practice. Perspectives on Psychological Science 15(2):384–396. 10.1177/1745691619896607 32069168 10.1177/1745691619896607

[CR54] R Core Team (2023). R: A language and environment for statistical computing. Vienna, Austria: R Foundation for Statistical Computing. https://www.R-project.org/

[CR55] Riglin L, Wootton RE, Thapar AK, Livingston LA, Langley K, Collishaw S, Tagg J, Smith GD, Stergiakouli E, Tilling K, Thapar A (2021) Variable emergence of autism spectrum disorder symptoms from childhood to early adulthood. Am J Psychiatry 178(8):752–760. 10.1176/appi.ajp.2020.20071119 33900814 10.1176/appi.ajp.2020.20071119 PMC7611492

[CR56] Scheeren AM, de Rosnay M, Koot HM, Begeer S (2013) Rethinking theory of mind in high-functioning autism spectrum disorder. J Child Psychol Psychiatry 54(6):628–635 23072222 10.1111/jcpp.12007

[CR57] Searle JR (1975) A Taxonomy of Illocutionary Acts. In: Gunderson K (ed) Language, Mind and Knowledge. University of Minnesota Press, pp 344–369

[CR58] Shatz M (1978a) Children’s comprehension of their mothers’ question-directives. J Child Lang 5(1):39–46. 10.1017/S0305000900001926

[CR59] Shatz M (1978b) On the development of communicative understandings: an early strategy for interpreting and responding to messages. Cogn Psychol 10(3):271–301. 10.1016/0010-0285(78)90001-4

[CR60] Spekman NJ, Roth FP (1985) Preschool children’s comprehension and production of directive forms. J Psycholinguist Res 14(3):331–349. 10.1007/BF01068090

[CR61] Steele S, Joseph RM, Tager-Flusberg H (2003) Brief report: Developmental change in theory of mind abilities in children with autism. J Autism Dev Disord 33(4):461–467. 10.1023/a:1025075115100 12959426 10.1023/a:1025075115100

[CR62] Trott S, Bergen B (2018) Individual differences in mentalizing capacity predict indirect request comprehension. Discourse Process 56(8):675–707. 10.1080/0163853X.2018.1548219

[CR63] Trott S, Bergen B (2020) When do comprehenders mentalize for pragmatic inference? Discourse Process 57(10):900–920. 10.1080/0163853X.2020.1822709

[CR64] Troyb E, Rosenthal M, Eigsti IM, Kelley E, Tyson K, Orinstein A, Barton M, Fein D (2014) Executive functioning in individuals with a history of ASDs who have achieved optimal outcomes. Child Neuropsychol J Normal Abnormal Develop Childhood Adolesc 20(4):378–397. 10.1080/09297049.2013.799644 10.1080/09297049.2013.799644 PMC3902134 23731181

[CR65] van Ackeren MJ, Casasanto D, Bekkering H, Hagoort P, Rueschemeyer SA (2012) Pragmatics in action: indirect requests engage theory of mind areas and the cortical motor network. J Cogn Neurosci 24(11):2237–2247. 10.1162/jocn_a_00274 22849399 10.1162/jocn_a_00274

[CR66] White S, Hill E, Happé F, Frith U (2009) Revisiting the strange stories: revealing mentalizing impairments in autism. Child Dev 80(4):1097–1117. 10.1111/j.1467-8624.2009.01319.x 19630896 10.1111/j.1467-8624.2009.01319.x

[CR67] Wimmer H, Perner J (1983) Beliefs about beliefs: representation and constraining function of wrong beliefs in young children’s understanding of deception. Cognition 13(1):103–128. 10.1016/0010-0277(83)90004-5 6681741 10.1016/0010-0277(83)90004-5

